# Metabolic and Microbiome Alterations Following the Enrichment of a High-Fat Diet With High Oleic Acid Peanuts Versus the Traditional Peanuts Cultivar in Mice

**DOI:** 10.3389/fnut.2022.823756

**Published:** 2022-06-15

**Authors:** Sarit Anavi-Cohen, Gil Zandani, Nina Tsybina-Shimshilashvili, Ran Hovav, Noa Sela, Abraham Nyska, Zecharia Madar

**Affiliations:** ^1^Peres Academic Center, Rehovot, Israel; ^2^Faculty of Agriculture, Food and Environment, The Hebrew University of Jerusalem, Rehovot, Israel; ^3^Department of Field Crops and Vegetables Research, Plant Sciences Institute, Agricultural Research Organization, Beit Dagan, Israel; ^4^Department of Plant Pathology and Weed Research, The Volcani Center, Rishon LeZion, Israel; ^5^Sackler School of Medicine, Tel Aviv University, Tel Aviv, Israel

**Keywords:** peanuts, inflammation, NAFLD, gut microbiome, oleic acid

## Abstract

A new Israeli-developed peanut cultivar, “Hanoch-Oleic” (HO), uniquely contains enlarged oleic acid contents and was designed to confer additional beneficial effects over the traditional cultivar, “Hanoch” (HN). This work elucidates metabolic changes and microbiota adaptations elicited by HO addition to a high-fat diet (HFD). Male C57BL/6 mice were fed for 18 weeks with a normal diet or a HFD with/without the addition of HN (HFDh) or HO (HFDo). Body-weight did not differ between HFD-fed mice groups, while liver and adipose weight were elevated in the HFDh and HFD groups, respectively. Insulin-sensitivity (IS) was also decreased in these groups, though to a much greater extent in the traditional peanuts-fed group. Modifications in lipids metabolism were evident by the addition of peanuts to a HFD. Liver inflammation seems to return to normal only in HFDh. Peanuts promoted an increase in α-diversity, with HFDo exhibiting changes in the abundance of microbiota that is primarily associated with ameliorated gut health and barrier capacity. In conclusion, the HO cultivar appears to be metabolically superior to the traditional peanut cultivar and was associated with an improved inflammatory state and microbial profile. Nevertheless, IS-negative effects reinforced by peanuts addition, predominantly NH, need to be comprehensively defined.

## Introduction

The World Health Organization defines obesity (and overweight) as an “abnormal or excessive fat accumulation that presents a risk to health” (1). Non-alcoholic fatty liver disease (NAFLD) is a comorbidity usually, though not always, associated with obesity. NAFLD is currently considered one of the most common forms of chronic liver disease worldwide and encompasses a wide spectrum of pathologies starting from simple steatosis through non-alcoholic steatohepatitis which may further progress to comprehensive fibrosis, cirrhosis, and hepatocellular carcinoma. NAFLD is prominently related to other metabolic alterations such as insulin resistance (IR), and a reciprocal relationship has been demonstrated between this disease and type 2 diabetes (2).

Obesity is also often manifested by IR and chronic low-grade inflammation. Adipose tissue has been implicated as an imperative participant in the establishment of systemic inflammation in the setting of obesity. Pro-inflammatory cytokines, other adipokines, along free fatty acids (FFA) secreted from adipose tissue may trigger and/or aggravate the development of IR and other pathological repercussions (2).

Peanuts are a dietary source of many nutrients and other compounds including proteins, fibers, vitamins (E, riboflavin, and folate), minerals (niacin, magnesium, selenium, manganese), and the well-acknowledged antioxidant polyphenols (3, 4). Fat constitutes the foremost macronutrient in peanuts and peanuts products, e.g., peanut butter and peanut oil. Unsaturated fatty acids comprise the vast majority of this fat, accounting for about 80% of all fats with half consisting of monounsaturated fatty acids (MUFAs) and the rest ∼30% of polyunsaturated fatty acids (PUFA). Hanoch (HN) is the traditional cultivar, also referred to as the conventional peanut, being used for industrial purposes. Given the encouraging metabolic and health-promoting effects attributed to oleic acid (5) a new peanut type was developed by the Israeli Agricultural Research Organization back in 2020 and was called “Hanoch-Oleic” (HO). With the goal of promoting human health, this improved cultivar is particularly characterized by augmented amounts of oleic acid which represents 82.7% of all fatty acids, compared to 55% in HN cultivar.

The profound influence exerted by the gut microbiome on human health is well recognized and has been established by numerous data. Diet and the metabolic state of the individual are interlinked with changes in the composition and activity of the gut microbiome. Compelling evidence has highlighted the mutuality that exists between diet, microbiome, and health, and this area has become the center of attention and a subject of ongoing research (6–8). Several studies demonstrated nut consumption affects gut microbiota composition is several taxonomical levels (9–11). However, the influence of peanuts specifically is largely obscure, with only a few studies conducted on this subject (12, 13). Moreover, the impact of HO peanuts cultivar consumption on the gut microbiome is yet to be tested. Thus, estimation of peanuts’ effects, especially those with a high oleic acid content, on the bacterial population in the gut is extremely desired.

The present work aimed to define the effects of habitual consumption of the traditional peanut cultivar, HN, and the new high-oleic peanut cultivar, HO, as an integral part of diet-induced obesity. Specifically, their role on the liver, adipose tissue, and gut microbiota was evaluated.

## Materials and Methods

### Experimental Animals and Diets

All animal experiments were done according to the rules of ethics of the Hebrew University of Jerusalem, Israel, and were approved by Institutional Animal Care Ethics Committee. All mice were maintained at 22 ± 2°C, controlled moisture of 60%, and in a 12 h light/12 h dark cycle with *ad libitum* access to food and water. After a 3-day acclimation period of standard rodent food, the food was replaced by the experimental diets. Thirty-two male 7–8 week old mice (C57BL/6J), were randomly divided into four groups (*n* = 8 per group). Experimental groups were as follows: (1) normal-diet (ND), (2) HFD, (3) HFD with “Hanoch” peanuts (HFDh), w/w 4%, (4) HFD with high oleic acid “Hanoch-Oleic” peanuts (HFDo), w/w 4%. Peanuts and diet composition are elaborated in Tables 1, 2. Diets were given for 18 weeks. Bodyweight was recorded once a week, and food intake was measured three times a week.

**TABLE 1:** Seeds composition of the two peanut varieties.

	Peanut variety	
	
Compounds	Hanoch (HN)*	Hanoch-Oleic (HO)*
Carbohydrates %	20.9	21.5
Protein %	23.5	22.6
Fat %	49.5	48.7
C16:0 (palmitic acid) %	8.4	4.99
C18:0 (stearic acid) %	3.23	2.03
C18:1n9 *cis* (oleic acid) %	55.28	82.68
C18:2n6 (linoleic acid) %	25.01	2.08

*HN, conventional peanut strain; HO, high oleic peanut strain.

**TABLE 2:** Diet consumption.

	Experiment 1			
	
Ingredients	ND*	HFD	HFDh	HFDo
Casein (gr)	21	26,5	25,48	25,48
L-Methionine (gr)	0,3	0,4	0,38	0,38
Corn starch (gr)	50	–	–	–
Maltodextrin (gr)	10	16	15,38	15,38
Anhydrous milkfat (gr)	2	–	–	–
Sucrose (gr)	3,9	9	8,65	8,65
Cellulose (gr)	3,5	6,6	6,35	6,35
Soybean oil (gr)	2	3	2,88	2,88
Lard (gr)	2	31	29,81	29,81
Peanut (gr)	–	–	3,85	3,85
Mineral mix (gr)	3,5	5,1	5,1	5,1
Vitamin mix (gr)	1,5	2,1	2,1	2,1
Choline chloride (gr)	0,3	0,3	0,3	0,3
BHT (gr)	0,014	0,014	0,014	0,014
Total (gr)	100	100	100	100
Total (kcal)	394,89	514	518,2	517,9
Protein (%)	21,58	21	21,13	21,08
Carbohydrate (%)	64,75	19,46	19,52	19,55
Fat (%)	13,68	59,53	59,07	59,04

*ND, normal diet; HFD, high fat diet; HFDh, high fat diet plus 4% (w/w) of Hanoch; HFDo, high fat diet plus 4% (w/w) of Hanoch-Oleic.

### Oral Glucose Tolerance Test

On the 16th week, an oral glucose tolerance test (OGTT) was performed in overnight (12 h) fasted mice as previously described (14).

### Animal Sacrifice and Organ Collection

At the end of the 18th week, the mice were fasted for 12 h, weighted, and randomly eliminated by isoflurane USP inhalation. Blood was drawn from the vena cava. Liver and adipose tissues were removed, weighed, placed in liquid nitrogen, and stored at –80°C. A small liver sample was also embedded in 4% formaldehyde. The cecum content was collected and stored at –80°C for microbiota analysis.

### Plasma Analysis

Blood liver enzymes and lipid profile analysis were conducted by American Laboratory (Herzliya, Israel). Plasma insulin levels were determined using an Insulin Rat/Mouse ELISA Kit (Cat. #EZRMI–13K). Homeostatic model assessment for insulin resistance (HOMA-IR) index was calculated as previously described (14). Plasma FFA was determined using an Abcam Free Fatty Acids Quantification Assay Kit (ab65341) according to the manufacturer’s instructions.

### Hepatic Triglyceride Levels and Histological Examination

Liver triglyceride levels were measured using the Triglyceride Quantification Assay Kit (Abcam, ab-65336), according to the manufacturer’s instructions. Histological slides were prepared by Patholab (Rehovot, Israel). The tissues were embedded in paraffin, and serial sections (3–5 μm thick) were cut from each block and stained with hematoxylin and eosin (H&E) as previously described (12).

### Western Blot Analysis

Tissues were extracted with lysis buffer, and the protein concentration was determined by the Bradford method followed by western blot. Blots were incubated at 4°C overnight with the primary antibodies: anti–adenosine monophosphate–activated protein kinase (AMPK) #2532; pAMPK, #2531; acetyl-CoA carboxylase (ACC), #3662; pACC #3661; CREB, p-CREB, inducible nitric oxide synthase (iNOS); and cluster of differentiation 36 (CD36), ab124515 ABCAM. The membranes were washed and then incubated with secondary goat antibodies (Jackson ImmunoResearch Laboratories, West Grove, PA, United States). The immune reaction was detected by enhanced chemiluminescence, with bands being quantified by densitometry and expressed as arbitrary units. An unspecific band out of the total protein (Ponceau) was used as housekeeping protein.

### Quantitative Real-Time Polymerase Chain Reaction

Total RNA was isolated from tissues using TRI Reagent (Sigma-Aldrich, Rehovot, Israel) according to the manufacturer’s protocol. Complementary DNA was prepared with the High-Capacity cDNA Reverse Transcription Kit (Quanta, BioSciences, Gaithersburg, MD, United States). Real-time polymerase chain reaction (PCR) was performed using the 7300 Real-Time PCR System (Applied Biosystems, Foster City, CA, United States), with specific primers. Quantitative changes in gene expression were determined by normalizing to 18S. Primer sequences are listed in Table 3.

**TABLE 3:** Primers sequences.

Name	Reverse	Forward
18s	5′-CCTCAGTTCCGAAAACCAAC-3′	5′-ACCGCAGCTAGGAATAATGG-3′
Fasn	5′-GGTCGTTTCTCCATTAAATTCTCAT-3′	5′-CTAGAAACTTTCCCAGAAATCTTCC-3′
SREBP-1c	5′-TAGATGGTGGCTGCTGAGTG-3′	5′-GATCAAAGAGGAGCCAGTGC-3′
TNFα	5′-CCACAAGCAGGAATGAGAAGA-3′	5′-ACGTGGAACTGGCAGAAGAG-3′
HSL	5′-TGCCCAGGAGTGTGTCTGAG-3′	5′-AGGACACCTTGGCTTGAGCG-3′
ATGL	5′-GGTTCAGTAGGCCATTCCTC-3′	5′-GTGCAAACAGGGCTACAGAG-3′
CPT-1	5′-CAGCGAGTAGCGCATAGTCA-3′	5′-TGAGTGGCGTCCTCTTTGG-3′
IL-6	5′-TGCAAGTGCATCATCGTTGT-3′	5′-ACTTCACAAGTCGGAGGCTTAAT-3′
PPARγ	5′-CAGCTTCTCCTTCTCGGCCT-3′	5′-CACAATGCCATCAGGTTTGG-3′
PGC-1α	5′-AGAGCAAGAAGGCGACACAT-3′	5′-AACAAGCACTTCGGTCATCC-3′
PPAR α	5′-CTGCGCATGCTCCGTG-3′	5′-CTTCCCAAAGCTCCTTCAAAAA-3′

*18S, 18S ribosomal RNA; Fasn, fatty acid synthase gene; SREBP-1c, sterol regulatory element-binding transcription factor 1; TNFα, tumor necrosis factor alpha; HSL, hormone sensitive lipase; ATGl, adipose triglyceride lipase; CPT-1, carnitine palmitoyl transferase I; IL-6, interleukin 6; PPARγ, peroxisome proliferator-activated receptor gamma; PGC-1 α, peroxisome proliferator-activated receptor gamma coactivator 1-alpha; PPAR α, peroxisome proliferator-activated receptor alpha.*

### Gut Microbiota Analysis

The effects of each diet on the bacterial population in the gut microbiome were examined with the analysis of the prokaryotic 16S ribosomal RNA gene (16S rRNA), using a two-step PCR-based method for preparing samples for sequencing the variable V3 and V4 regions of the 16S rRNA gene (12). The paired-end sequences were processed, paired, and trimmed, and low-quality reads were removed using the “QIIME2” software package. Chimeric sequences were filtered using the chimera-removal function of UCHIME (15), implemented within “QIIME2.” Illumina sequence denoising was done *via* DADA2 implemented in QIIME2 (16). Sequences with 97% similarity were assigned to the same operational taxonomic units (OTU). Chloroplast and mitochondrial 16S rRNA sequences, as well as all OTUs represented by fewer than 10 sequences, were removed from the OTU count table. The count data were rarefied to avoid variable library size bias (17). OTUs of representative sequences at a similarity of 97% and their relative abundances were used to calculate and analyze rarefaction curves. Bacterial richness and diversity within samples were classified by alpha diversity (Pielou’s index, observed-species indices, and Shannon index).

### Statistical Analysis

Statistical analysis was performed using a one-way analysis of variance (one-way ANOVA) followed by the Tukey–Kramer HSD *post hoc* test. The results are presented as means ± standard error (SEM). For all analyses, the JMP 14 Pro Software Suites (SAS Institute, Cary, NC, United States) was used. All *p* values were calculated using these two tests. *p* Values less than 0.05 were considered statistically significant.

## Results

### Effect of Peanuts Addition to a High-Fat Diet on Mice Weight, Food Intake, and Liver and Adipose Tissue Weight

**FIGURE 1:**
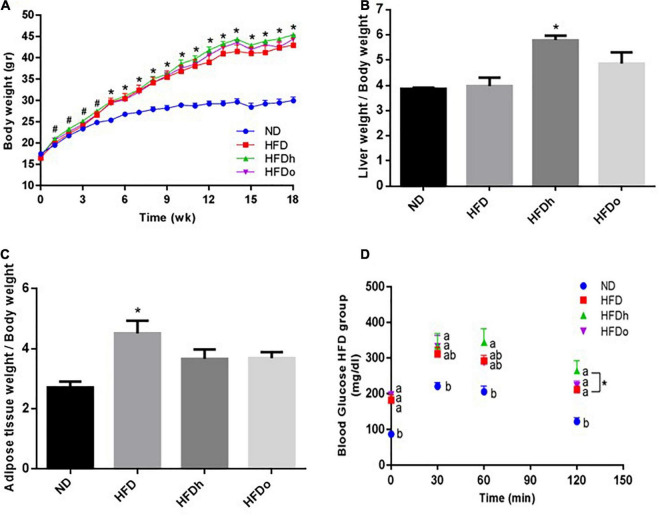
The effect of diets on body, liver and adipose tissue weight and glucose tolerance. Mice consumed either a normal diet (ND), a high-fat diet (HFD), a high-fat diet plus 4% (w/w) of HN (HFDh) or HO (HFDo) peanuts for 18 weeks. Body weight **(A)**, liver weight to body weight **(B)**, and adipose tissue weight to body weight **(C)** ratios were measured. Two weeks before the end of the experimental period, an oral glucose tolerance test (OGTT) was carried out **(D)**. A Tukey-Kramer HSD post hoc test and the Student’s *T*-test were performed. The values presented are mean ± SE (*n* = 8). Different letters indicate statistical variance at a significance level of *p* < 0.05.

As shown in Figure 1A, at the end of the experiment, body weight was profoundly higher in all HFD-fed groups compared with the control (ND). In comparison with the control group, liver-to- body weight ratio was elevated only in the group that was supplemented with the regular peanut cultivar whereas a significant increase in adipose tissue weight-to-body weight ratio was found merely in the HFD group (Figures 1B,C). Measurement of hepatic TG levels and histological staining further corroborated similar TG/lipid accumulation in the livers of all HFD-fed groups, with solely the HFDh group demonstrating significantly increased values above those of the control in the former analysis (Supplementary Figures 1A,B).

### Effects of Peanuts Addition to a High-Fat Diet on Blood Biochemistry Profile

Blood TG levels were lower in the HFDh and HFDo groups compared to the ND group, while no significant differences were found between the HFD-fed groups. Total blood cholesterol levels were significantly elevated in all HFD-groups, with the HFDo group exhibiting higher levels compared to HFD alone. HDL cholesterol was markedly augmented in all HFD groups. FFA blood levels only differed between HFD and HFDh groups, with levels being lower in the former. Compared with the control, blood levels of the liver enzymes AST and ALT were elevated in the HFD groups that were supplemented with peanuts, while those of ALT were significantly and similarly elevated in all HFD-fed groups. No change was observed in ALP levels (Table 4).

**TABLE 4:** Blood biochemistry profile.

	Groups			
	
Parameters	ND	HFD	HFDh	HFDo
Triglycerides (mg/dl)	101.67 ± 9.89^a^	80.75 ± 5.31^ab^	72.38 ± 2.22^b^	65.29 ± 4.89^b^
Total cholesterol (mg/dl)	124.5 ± 5.94^c^	197.38 ± 15.02^b^	229 ± 11.15^ab^	245 ± 8.79^a^
HDL cholesterol (mg/dl)	99.77 ± 3.97^b^	147.64 ± 10.38^a^	163.79 ± 6.37^a^	175.84 ± 5.87^a^
FFA (nmol/μl)	0.29 ± 0.01^ab^	0.24 ± 0.01^b^	0.30 ± 0.02^a^	0.27 ± 0.01^ab^
AST (IU/L)	52.6 ± 5.18^c^	88.71 ± 10.86^bc^	101 ± 8.79^ab^	134.86 ± 11.17^a^
ALT (IU/L)	31.2 ± 7.56^b^	112.13 ± 19.44^a^	114.25 ± 13.52^a^	158.38 ± 11.73^a^
ALP (IU/L)	69.67 ± 4.18	70.88 ± 7.07	76.88 ± 4.51	76.25 ± 4.5
Insulin (mmol/l)	6.97 ± 0.65^b^	10.51 ± 0.34^b^	14.52 ± 0.99^a^	14.92 ± 1.29^a^
HOMA-IR index	0.51 ± 0.07^c^	4.04 ± 0.68^c^	15.74 ± 1.61^a^	9.42 ± 1.65^b^

*The effect of diets on lipid profiles, free fatty acid (FFA) blood levels, liver enzymes, insulin level, and HOMA-IR Index. Mice consumed either a normal diet (ND), a high-fat diet (HFD), a high-fat diet plus 4% (w/w) of HN (HFDh) or HO (HFDo) peanuts for 18 weeks. At the end of the experiment, plasma triglycerides, cholesterol, HDL-cholesterol (high-density lipoprotein cholesterol), alkaline phosphatase (AST), glutamic-pyruvic transaminase (ALT), glutamic oxaloacetic transaminase (ALP), FFA levels, and insulin levels were measured in the plasma (n = 8). HOMA-IR index was calculated by using the formula: [Fasting insulin concentration (microU/ml) × Glucose concentration (mg/dl)/405]. A Tukey–Kramer HSD post hoc statistical test was performed. The value presented are mean ± SE (n = 8). Different letters indicate statistical variance at a significant level of p < 0.05.*

### Effects of Peanuts Addition to a High-Fat Diet on the Glycemic Response, Homeostatic Model Assessment for Insulin Resistance Index, and Liver Gluconeogenesis

Oral glucose tolerance test was carried out to assess the effect of the diets on the glycemic response. The ND group had the lowest blood glucose levels throughout the test (Figure 1D). At the last time point (120 min), a noteworthy difference was observed between the HFDh and HFD groups, with blood glucose being greater in the former. No significant changes were found between the groups in ΔOGTT or AUC analyses (data not shown).

Fasting plasma insulin concentrations at the end of the experiment were higher in the HFDh and HFDo groups than in the HFD and ND groups. Consequently, HOMA-IR Index, a surrogate measure of insulin resistance, was profoundly greater in those HFD groups that were supplemented with peanuts (Table 4).

**FIGURE 2:**
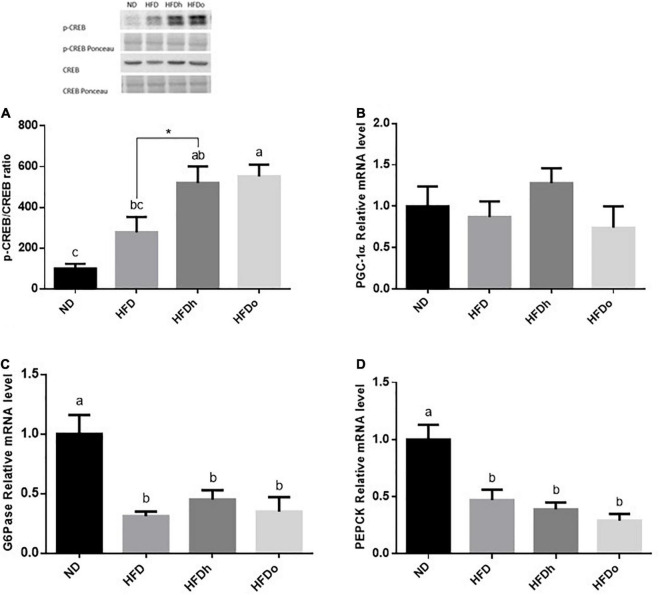
The effect of diets on key players that participate in carbohydrate metabolism in the liver. Mice consumed either a normal diet (ND), a high-fat diet (HFD), a high-fat diet plus 4% (w/w) HN (HFDh) or HO (HFDo) peanuts for 18 weeks. The p-CREB/CREB protein ratio **(A)** was measured using Western blot where an unspecific band of total protein (Ponceau) was used as a control protein. The PGC-1α **(B)**, G6Pase **(C)**, and PEPCK **(D)** gene expressions were measured at the transcription level (RT-PCR), and the results normalized to the 18S gene expression. A Tukey’s Kramer post-hos test and Student’s *T*-test were performed. The values displayed are mean ± standard error. Columns marked with different letters indicate statistically significant variances at *p* < 0.05.

The effect of diets on liver gluconeogenesis was assessed by measuring the expression of key players in this metabolic pathway. The rate of CREB activation, measured as phosphorylated CREB at Ser133/total CREB ratio, was markedly and significantly increased by the consumption of HFD supplemented with peanuts (Figure 2A). However, this increase was not paralleled to a similar gene expression pattern, in which PGC-1α, G6Pase, and PEPCK mRNA levels were unaffected or even downregulated by HFD with or without peanuts addition (Figures 2B–D).

### Effects of Peanuts Addition to a High-Fat Diet on Liver Lipid Metabolism and Related Regulators

**FIGURE 3:**
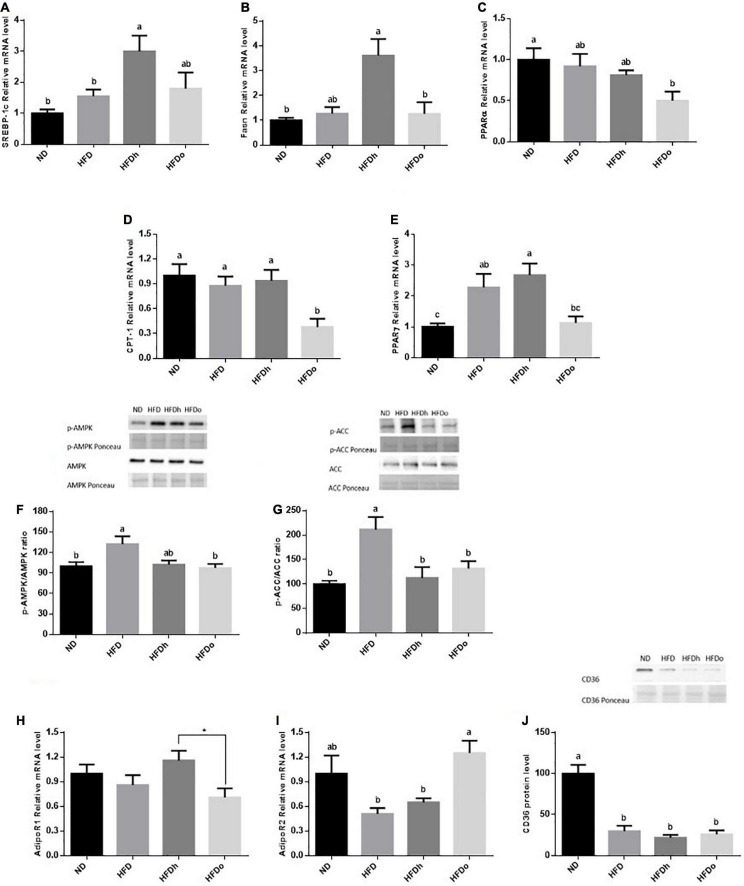
The effect of diets on key players that participate and/or regulate lipid metabolism in the liver. Mice consumed either a normal diet (ND), a high-fat diet (HFD), a high-fat diet plus 4% (w/w) HN (HFDh) or HO (HFDo) peanuts for 18 weeks. The SREBP-1c **(A)**, Fasn **(B)**, PPARα **(C)**, CPT-1 **(D)**, PPARγ **(E)**, AdipoR1 **(H)** and AdipoR2 **(I)** gene expressions were measured at the transcription level (RT-PCR), and the results normalized to the 18S gene expression. The p-AMPK/AMPK protein ratio **(F)**, p-ACC/ACC protein ratio **(G)**, and CD36 protein levels **(J)** were measured using Western blots where an unspecific band of total protein (Ponceau) was used as a control protein. A Tukey’s Kramer post-hos test and Student’s *T*-test were performed. The values displayed are mean ± standard error. Columns marked with different letters indicate statistically significant variances at *p* < 0.05.

Liver lipid metabolism was examined by measuring the expression of factors participating in *de novo* lipogenesis as well as lipid oxidation. SREBP-1c and Fasn gene expression was significantly elevated in the HFDh group compared to the other groups (Figures 3A,B). PPARα and CPT-1 gene expression was attenuated merely in the HFDo group, compared to the control. Furthermore, whereas HFD and HFDh exhibited a significant increase in PPARγ expression levels, HFDo was also the only group whose this parameter was similar to that of the control (Figures 3C–E). Additional analyses were carried out to clarify whether the AMPK pathway was affected by the diets combination. While AMPK activation was amplified in the HFD group, as assessed by phospho-to-total AMPK and ACC ratios, this activation was significantly abolished by the addition of peanuts to HFD (Figures 3F,G). Despite being an important activator of the AMPK pathway, adiponectin receptors expression did not correspond with AMPK activation. Characterization of these receptors revealed an inverse expression pattern between AdipoR1 and AdipoR2. Whereas AdipoR1 expression was significantly higher in HFDh relatively to HFDo, the complete opposite result was registered in AdipoR2 expression, i.e., greater levels in the HFDo group compared to the HFDh group (Figures 3H,I). Finally, under the HFD regime, FFA uptake into liver cells appears to be diminished as reflected by the profound decrease observed in CD36 protein levels in all HFD-fed groups (Figure 3J).

### The Effect of Peanuts Addition to a High-Fat Diet on Liver Inflammation

**FIGURE 4:**
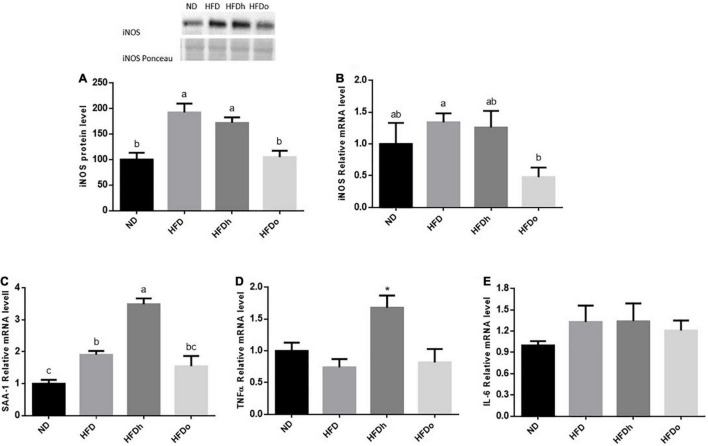
The effect of diets on protein and inflammatory genes expression in the liver. Mice consumed either a normal diet (ND), a high-fat diet (HFD), a high-fat diet plus 4% (w/w) HN (HFDh) or HO (HFDo) peanuts for 18 weeks. The protein expression of iNOS **(A)** was measured using Western blot where an unspecific band of total protein (Ponceau) was used as a control protein. The gene expressions of iNOS **(B)**, SAA-1 **(C)**, TNFα **(D)**, and IL-6 **(E)** were measured at the transcription level (RT-PCR), and the results were normalized to the 18S gene expression. A Tukey’s Kramer post-hos test and ANOVA (Dunnett’s) statistical test were performed. The values displayed are mean ± standard error. Columns marked with different letters indicate statistically significant variances at *p* < 0.05.

iNOS appears to be induced in the liver by the consumption of HFD, as seen by the increase in this enzyme protein levels. Although iNOS expression was not significantly altered by this diet, a similar trend was found. Importantly, the addition of the oleic-rich peanut to HFD prevented iNOS induction at both, protein and expression, levels (Figures 4A,B). A similar pattern was found in SAA-1. Accordingly, whereas HFD promoted this gene expression, the increase was insignificant by the addition of oleic-rich peanuts (Figure 4C). Interestingly, HFD supplemented with the other, “traditional” peanut strain, not only failed to decrease the inflammatory state of the liver but rather seems to further enhance it. Indeed, the HFDh group exhibited the highest SSA-1 expression levels, greater than the levels found following the consumption of HFD alone. Consistently, whereas HFD did not affect TNFα nor IL-6 expression, the expression of the former was induced in the HFDh group (Figures 4D,E).

### Effects of Peanuts Addition to a High-Fat Diet on Adipose Lipid Metabolism and Inflammation in Adipose Tissue

**FIGURE 5:**
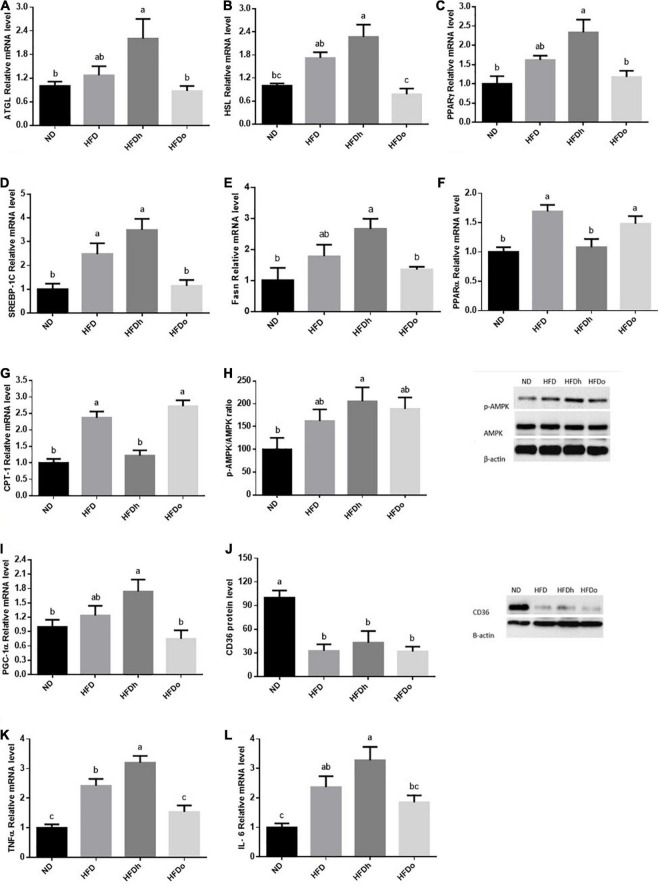
The effect of diets on key players that participate in fat metabolism and inflammation in adipose tissue. Mice consumed either a normal diet (ND), a high-fat diet (HFD), a high-fat diet plus 4% (w/w) of HN (HFDh) or HO (HFDo) peanuts for 18 weeks. The ATGL **(A)**, HSL **(B)**, PPARγ **(C)**, SREBP-1c **(D)**, Fasn **(E)**, PPARα **(F)**, CPT-1 **(G)**, PGC-1α **(I)**, Il-6 **(K)** and TNFα **(L)** genes expressions were measured at the transcription level (RT-PCR), and the results normalized to the 18S gene expression. The p-AMPK/AMPK ratio **(H)** and CD36 **(J)** protein levels were measured using Western blots where β-actin was used as a control protein. A Tukey’s Kramer post-hos test was performed. The values displayed are mean ± standard error. Columns marked with different letters indicate statistically significant variances at *p* < 0.05.

High-fat diet insignificantly tended to increase the expression of the lipolytic enzymes, ATGL and HSL. However, while the expression of both genes was further increased by the addition of regular peanuts to HFD, changes were completely blunted by the addition of oleic-rich peanuts to the HFD (Figures 5A,B). To examine the effect of HFD with or without peanuts addition on adipose tissue lipogenesis, PPARγ, SREBP-1c, and Fasn gene expression were measured. Whereas only SREBP-1c expression was positively induced by HFD, the expression of all tested genes was significantly upregulated in the HFDh group, compared to the ND control. Conversely, the HFDo group did not exhibit any alteration in the expression of these genes and displayed equal levels as those of the ND group (Figures 5C–E).

The expression pattern of PPARα and CPT-1, which participate in FFA oxidation, was similar. While the consumption of HFD and HFDo enhanced the expression of these genes, no marked change was found following the consumption of HFDh (Figures 5F,G). Compared to the ND group, AMPK activation was significantly increased in the HFDh group whereas a trend toward higher levels was noticed in HFD and HFDo groups (Figure 5H). A rather similar pattern was observed with PGC-1α gene expression. However, this measure significantly differed between HFDh and HFDo groups, with PGC-1α being greater in the former (Figure 5I). CD36 protein levels were profoundly decreased by the consumption of HFD regardless of the addition of peanuts to this diet (Figure 5J).

Adipose tissue inflammatory state is known to impact the whole-body metabolism. Thus, to further gain insight into the effects of the different peanut strains, key inflammatory markers were assessed. Consumption of HFD considerably elevated the pro-inflammatory cytokines TNFα and IL-6 expression. TNFα and IL-6 expression was significantly or tended to further increase in the HFDh group, respectively. Conversely, these gene expression levels were not significantly induced by the addition of high-oleic peanuts to HFD compared to the ND control (Figures 5K,L).

### Effect of Peanuts Addition to a High-Fat Diet on Intestinal Microbiota

The effects of diets on microbiota species richness and diversity (observed OUT’s and Shannon Index, respectively) were evaluated as well. Diversity, which explicitly represents the integration of species richness with evenness into a distinct value (18), was found to be upraised by peanuts consumption, regardless of the cultivar been used. Species abundance *per se*, the evenness component of diversity, as measured by the Pielou’s index, was marginally increased by HFD consumption with additional effect being observed only in the HFDh group (Table 5).

**TABLE 5:** Taxonomical levels and diversity indexes.

	ND	HFD	HFDh	HFDo
**α diversity**				
Observed OUT’s	141.80 ± 3.20	133.75 ± 4.67	162.80 ± 7.69^#^	168.00 ± 12.63^#^
Shannon index	6.00 ± 0.07	6.15 ± 0.06	6.61 ± 0.12^*^,^#^	6.55 ± 0.15^*^,^#^
Pielou’s index	0.84 ± 0.01	0.87 ± 0.01*	0.90 ± 0.01^*^,^#^	0.89 ± 0.01*
**Phylum level**				
Actinobacteria	1.30 ± 0.21	0.49 ± 0.12*	0.67 ± 0.20*	0.25 ± 0.06*
Bacteroidetes	51.70 ± 2.69	34.21 ± 2.24*	30.03 ± 1.89*	32.03 ± 2.14*
Deferribacteres	1.16 ± 0.09	1.56 ± 0.28	0.93 ± 0.13^#^	0.95 ± 0.25^#^
Firmicutes	39.75 ± 2.86	52.83 ± 1.93*	58.30 ± 2.29*	58.82 ± 3.85*
Proteobacteria	4.36 ± 0.52	10.10 ± 1.70*	8.50 ± 0.94	8.63 ± 1.30*
Tenericutes	0.34 ± 0.11	0.23 ± 0.08	0.74 ± 0.18^*^,^#^	0.41 ± 0.06
Verrucomicrobia	0.96 ± 0.03	0.00 ± 0.00^#^	0.48 ± 0.36	0.00 ± 0.00^#^
F/B ratio	0.79 ± 0.10	1.57 ± 0.16*	1.98 ± 0.19*	1.90 ± 0.26*
**Class level**				
Coriobacteriia	0.65 ± 0.12	0.50 ± 0.12	0.58 ± 0.18^$^	0.20 ± 0.03*
Bacteroidia	51.69 ± 2.69	34.21 ± 2.24*	30.03 ± 1.89*	32.03 ± 2.14*
Deferribacteres	1.16 ± 0.10	1.56 ± 0.28	0.93 ± 0.13^#^	0.95 ± 0.25^#^
Clostridia	36.84 ± 2.93	50.43 ± 2.29*	54.69 ± 2.09*	56.98 ± 3.78*
Erysipelotrichi	1.64 ± 0.11	1.48 ± 0.53	1.96 ± 0.51^$^	0.26 ± 0.15^*^,^#^
Alphaproteobacteria	0.56 ± 0.18	0.16 ± 0.03*	0.10 ± 0.06*	0.45 ± 0.13
Deltaproteobacteria	3.55 ± 0.46	9.70 ± 1.65*	8.15 ± 0.85*	9.28 ± 1.41*
Mollicutes	0.34 ± 0.11	0.23 ± 0.08	0.75 ± 0.18^*^,^#^	0.41 ± 0.06
Verrucomicrobiae	3.21 ± 0.51	0.00 ± 0.00*	0.48 ± 0.36	0.00 ± 0.00*
**Order level**				
Bifidobacteriales	0.65 ± 0.17	0.00 ± 0.00*	0.08 ± 0.05*	0.05 ± 0.05*
Coriobacteriales	0.65 ± 0.12	0.5 ± 0.12	0.58 ± 0.18^$^	0.20 ± 0.03*
Bacteroidales	51.69 ± 2.69	34.21 ± 2.24*	30.03 ± 1.89*	32.03 ± 2.14*
Deferribacterales	1.16 ± 0.10	1.56 ± 0.28	0.93 ± 0.13^#^	0.95 ± 0.25^#^
Clostridiales	36.84 ± 2.93	50.43 ± 2.29*	54.69 ± 2.09*	56.98 ± 3.77*
Erysipelotrichales	1.64 ± 0.11	1.48 ± 0.53	1.96 ± 0.51^$^	0.26 ± 0.15^*^,^#^
RF32	0.56 ± 0.18	0.16 ± 0.03*	0.10 ± 0.06*	0.45 ± 0.13
Desulfovibrionales	3.55 ± 0.46	9.70 ± 1.65*	8.15 ± 0.85*	9.28 ± 1.41*
Anaeroplasmatales	0.27 ± 0.09	0.03 ± 0.03	0.32 ± 0.14^#^,^[*$*]^	0.04 ± 0.03
RF39	0.07 ± 0.03	0.20 ± 0.08	0.43 ± 0.12*	0.37 ± 0.06*
Verrucomicrobiales	0.96 ± 0.31	0.00 ± 0.00*	0.48 ± 0.36	0.00 ± 0.00*
**Family level**				
Bifidobacteriaceae	0.65 ± 0.17	0.00 ± 0.00*	0.08 ± 0.05*	0.05 ± 0.05*
Coriobacteriaceae	0.65 ± 0.12	0.5 ± 0.12	0.58 ± 0.18^$^	0.20 ± 0.03*
Bacteroidaceae	6.98 ± 1.02	5.57 ± 1.09	2.10 ± 0.45^*^,^#^	3.48 ± 0.45*
Porphyromonadaceae	0.62 ± 0.13	0.45 ± 0.16	4.56 ± 1.63^*^,^#^	3.74 ± 1.83
Prevotellaceae	0.00 ± 0.00	0.01 ± 0.01	0.02 ± 0.01^$^	0.23 ± 0.08^*^,^#^
Rikenellaceae	5.32 ± 0.42	5.42 ± 0.26	7.69 ± 0.86^*^,^#^	7.73 ± 0.75^*^,^#^
S24-7	14.77 ± 1.23	7.31 ± 1.45*	8.43 ± 0.64*	7.74 ± 0.94*
Deferribacteraceae	1.16 ± 0.10	1.56 ± 0.28	0.93 ± 0.13^#^	0.95 ± 0.25^#^
Clostridiaceae	0.54 ± 0.09^#^	0.00 ± 0.00	0.59 ± 0.06^#^	0.41 ± 0.11^#^
Dehalobacteriaceae	0.26 ± 0.08	0.41 ± 0.07	0.55 ± 0.07*	0.48 ± 0.05*
Lachnospiraceae	4.47 ± 0.57	9.88 ± 0.96*	9.09 ± 0.45*	9.43 ± 0.56*
Peptococcaceae	0.18 ± 0.03	0.49 ± 0.08	0.43 ± 0.04	0.55 ± 0.13
Peptostreptococcaceae	0.00 ± 0.00	0.34 ± 0.13*	0.57 ± 0.04*	0.53 ± 0.11*
Ruminococcaceae	10.63 ± 0.97	14.59 ± 1.98	13.48 ± 0.99	16.94 ± 2.12*
Erysipelotrichaceae	1.64 ± 0.11	1.48 ± 0.53	1.96 ± 0.51^$^	0.26 ± 0.15^*^,^#^
Desulfovibrionaceae	3.55 ± 0.46	9.70 ± 1.65*	8.15 ± 0.85*	9.28 ± 1.41*
Anaeroplasmataceae	0.27 ± 0.09	0.03 ± 0.03	0.32 ± 0.14^#^,^[*$*]^	0.04 ± 0.03
Verrucomicrobiaceae	0.96 ± 0.31	0.00 ± 0.00*	0.48 ± 0.36	0.00 ± 0.00*
**Genus level**				
Bifidobacterium	0.65 ± 0.17	0.00 ± 0.00*	0.08 ± 0.05*	0.05 ± 0.05*
Adlercreutzia	0.31 ± 0.05	0.26 ± 0.05	0.18 ± 0.04*	0.19 ± 0.03
Bacteroides	6.98 ± 1.02	5.57 ± 1.09	2.10 ± 0.45^*^,^#^	3.48 ± 0.45*
Parabacteroides	0.62 ± 0.13	0.45 ± 0.16	4.56 ± 1.63^*^,^#^	3.74 ± 1.83
Prevotella	0.00 ± 0.00	0.01 ± 0.01	0.02 ± 0.01^$^	0.23 ± 0.08^*^,^#^
AF12	1.20 ± 0.22	1.17 ± 0.25	2.51 ± 0.56^*^,^#^	2.25 ± 0.36
Mucispirillum	1.16 ± 0.10	1.56 ± 0.28	0.93 ± 0.13^#^	0.95 ± 0.25^#^
Dehalobacterium	0.26 ± 0.08	0.41 ± 0.07	0.55 ± 0.07*	0.48 ± 0.05*
Coprococcus	0.38 ± 0.20	0.52 ± 0.20	0.79 ± 0.10	1.08 ± 0.21*
Ruminococcus	1.49 ± 0.12	2.28 ± 0.48*	1.97 ± 0.17	1.65 ± 0.16
Oscillospira	3.97 ± 0.43	6.42 ± 1.12*	4.38 ± 0.33	5.91 ± 0.77
Allobaculum	1.62 ± 0.11	1.45 ± 0.51	1.96 ± 0.51^$^	0.26 ± 0.15^*^,^#^
Bilophila	1.87 ± 0.47	7.09 ± 1.30*	3.52 ± 0.56^#^	5.61 ± 1.41*
Desulfovibrio	1.26 ± 0.14	1.90 ± 0.35	4.02 ± 0.74^*^,^#^	2.78 ± 0.30*
Anaeroplasma	0.27 ± 0.09	0.03 ± 0.03	0.32 ± 0.14^#^,^[*$*]^	0.04 ± 0.03
Akkermansia	0.96 ± 0.31	0.00 ± 0.00*	0.48 ± 0.36	0.00 ± 0.00*
**Species level**				
Acidifaciens	1.43 ± 0.24	0.91 ± 0.13	0.93 ± 0.17	0.66 ± 0.18*
Schaedleri	1.16 ± 0.10	1.56 ± 0.28	0.93 ± 0.13^#^	0.95 ± 0.25^#^
Gnavus	1.49 ± 0.12	2.28 ± 0.48*	1.97 ± 0.17	1.65 ± 0.16
Flavefaciens	0.45 ± 0.10	0.00 ± 0.00*	0.03 ± 0.02*	0.19 ± 0.09*
Muciniphila	0.96 ± 0.31	0.00 ± 0.00*	0.48 ± 0.36	0.00 ± 0.00*

The effect of diets on gut microbiota richness and diversity as well as microbiota composition at all taxonomic levels. Mice were fed with normal diet (ND), high fat diet (HFD), high fat diet plus 4% (w/w) of Hanoch (HFDh) or high fat diet plus 4% (w/w) of Hanoch-Oleic (HFDo) for 18 weeks. Values are expressed as mean ± SEM (n = 5). *p < 0.05 versus ND group; ^#^p < 0.05 versus HFD; ^$^p < 0.05 versus HFDo group.

Significant alterations in gut microbiota composition are elaborated in Table 5. At the phylum level, peanuts addition, as well as HFD, elicited profound changes in the two most dominant bacterial phyla, Bacteroidetes and Firmicutes, leading to decreased and decreased levels, respectively. Consequently, the F/B ratio was elevated by these treatments. Actinobacteria levels were decreased whereas those of Proteobacteria were elevated or tended to be higher in the HFD-fed group with or without peanuts addition. Deferribacteres presence was diminished in the groups where peanuts were added to HFD, regardless of the cultivar. Finally, Tenericutes abundance was abolished following the consumption of the HFD or HFDo group while no change was noted in the HFDh group. At the family level, Bifidobacteriales, Bacteroidales, RF32, and Verrucomicrobiales abundance was lower in all HFD-fed groups, except for the RF32 family in the HFDo group and Verrucomicrobiales family in the HFDh group. Conversely, greater quantities of Clostridiales and Desulfovibrionales were found in all HFD-fed groups. A marked decrease in the Erysipelotrichales family was observed in the group fed with HFD with the addition of high-oleic peanuts, compared with the rest of the groups. On the other hand, Anaeroplasmatales levels were higher in the HFDh group, compared to HFD and HFDo groups, with levels comparable to the control.

## Discussion

The present work elucidated several metabolic consequences driven by the incorporation of the new, predominantly oleic-rich, peanut cultivar HO to a HFD and further compared them to those obtained by the addition of the traditional cultivar, HN, to the same diet. This study further characterized the alterations in gut resident microbiota implemented by these dietary manipulations. Although the addition of peanuts did not abolish the irregularities emanating from the obesogenic diet, results posit HO constitutes the preferred type in metabolic terms and plausibly in microbiota adaptation.

Peanut addition, regardless of the type, under the setting of diet-induced obesity failed to mitigate the increase in body weight. However, under obesogenic conditions, not only is the amount of weight-gain important but also where it is stored. Under a HFD regime, the liver-to-bodyweight ratio was significantly elevated only in the group that was supplemented with the conventional peanut “Hanoch,” indicating ectopic fat accumulation in this group. The ratio between epididymal adipose tissue, a representative of the visceral fat pad in rodents (12–19) to-total body weight was comparable between ND and HFD-supplemented with peanuts but was markedly increased in the HFD alone, suggesting more fat stored as visceral fat. The lack of profound changes in the ratio between liver and epididymal fat pad to body weight in the HFDo group, despite an increase in body weight, may infer that this peanut cultivar is associated with a “healthier” pattern of weight gain.

Analysis of insulin regulation revealed enhanced IR when the conventional peanut cultivar was added to the HFD. When the high-oleic cultivar was added to the HFD, though IR in this group was higher than that of the HFD-fed group, IR was much less profound in this group than in the regular cultivar-supplemented group. Consist with this, in a previous study, the addition of traditional peanuts to a HFD ameliorated the AUC obtained from OGTT, though was associated with increased fasting insulin concentrations compared to HFD alone. Conversely, the addition of a different high-oleic cultivar used in that study did not elicit any extra deleterious effect on those of the HFD *per se* (12). The clinical outcomes attributed to long-term peanuts consumption have been investigated by several works (4). Concerning glycemic factors, results are conflicting. Although no change in fasting blood glucose was found in several works, others have reported a decrease or, alternatively, an increase in this parameter by incorporating peanuts into the diet (4). Similarly, blood insulin was either unchanged or increased following peanuts consumption (12). Regarding high-oleic peanuts specifically, Moreira et al. investigated the metabolic consequences of such a peanut cultivar during the consumption of HFD. These researchers found no advantage for the high-oleic peanuts on fasting blood glucose and insulin levels over the conventional cultivar. Yet, a moderated glycemic response following meal intake was observed (20, 21). These, along with the current findings, do not support the notion that peanuts with high-oleic content convey fundamental benefits and effects on blood glucose and/or insulin levels though they may be advantageous to/over the conventional cultivar under specific circumstances.

The mechanism by which the addition of peanuts negatively affected insulin sensitivity in the present work is not understood. However, under conditions of elevated oxidative stress, double-bond/s containing FA might be oxidized to yield harmful products (22). Supporting this notion, AST levels were significantly augmented in the peanuts-enriched groups. These findings may argue for the essential need for additional antioxidant compounds when obesogenic diets are fortified with unsaturated FA (23).

Abnormally activated GNG is well acknowledged as one of the processes that pathologically influence fasting blood glucose. GNG is controlled, among other mechanisms, at the transcriptional level through the transcription factor CREB, which is activated through its phosphorylation at ser133 residue. Activated CREB subsequently potentiates GNG by upregulating the expression of the key GNG enzymes PEPCK and G6Pase and the expression of the co-activator PGC-1α (24). Surprisingly, in the current study, CREB activation was not concomitant with a similar increase in the expression of pro/GNG genes. In fact, the mRNA levels of both GNG enzymes were downregulated in mice that were fed with HFDh, and HFDo despite a significant induction of CREB. Likewise, the tendency toward CREB activation in the HFD group was accompanied by a decrease rather than an increase in PEPCK and G6Pse gene expression. This lack of coherency is obscure. Yet, several mechanisms may be accounted for this phenomenon, including the disruption of the CREB/CRTC2 complex by insulin. Insulin is a robust negative regulator of GNG at several levels, including by mediating the interaction between activated CREB and small heterodimer partner–interacting leucine zipper protein (SMILE) instead of with CRTC2. CREB-SMILE complex is unable to induce PEPCK and G6Pase nor PGC-1alpha gene expression (25). Given that Insulin levels were or tended to be increased in the same mentioned groups and the ability of IR to be selective in the liver, it is posited that insulin can be the cause of the discrepancy between CREB activation and GNG-gene expression. Nevertheless, since the outcome of GNG, i.e., fasting blood glucose specifically, remained unaltered, it is presumed levels were counterbalanced by enhanced glycogenolysis or increased peripheral IR in HFD-fed groups.

Much attention has also been drawn to the impact of peanuts intake on lipidemia. Although not well established, it is consensually believed a reduction in TG and LDL cholesterol levels is among the health-promoting effects peanuts intake may carry out (3). In the present study, TG levels were lower while those of HDL were greater in all HFD-fed groups without any significant impact on peanuts addition. In accord, a recent meta-analysis failed to demonstrate a positive effect of peanuts consumption on blood TG levels.

Over the years, several works have appraised the usefulness of nuts, including peanuts, on liver fat concentration with the results being controversial (3). The results presented here demonstrate diverse outcomes can be derived depending on the dietary regime applied and/or peanut cultivar being used. As stated, under an obesogenic diet, the development of NAFLD was further promoted by the addition of conventional peanuts to HFD, as manifested by the liver to body weight ratio in this group. Inline, this group exhibited augmented expression of key players required for the implementation of *de novo* lipogenesis while those that govern fatty acids oxidation were unaffected. Altered adipose tissue lipid metabolism, lipolysis specifically, is among the salient factors contributing to the establishment of liver steatosis. ATGL and HSL expression in adipose tissue was significantly upregulated by the addition of traditional peanuts to this diet, suggesting enhanced adipocyte lipolytic capacity in this group which may subsequently contribute to ectopic fat accumulation in the liver. On the contrary, liver steatosis was more constrained in the group fed with the alternate, high-oleic peanuts cultivar, as seen by the insignificantly increased liver to body-weight ratio and hepatic TG contents. Lower hepatic and adipocytes lipogenic capacity concomitantly to lower adipocytes lipolysis and enhanced FFA oxidation may facilitate the discrepancy in liver-fat deposition between the two peanuts cultivars.

Anti–adenosine monophosphate–activated protein kinase is a crucial regulator known to orchestrate numerous signaling pathways involved in glucose and lipid metabolism. AMPK directs the cellular adjustment and facilitates the transition from anabolic to catabolic pathways, intending to restore energy homeostasis. A previous study has found enhanced AMPK activation following the exposure of myotube cells to oleate (26), purporting similar induction may be accountable for the advantages metabolic impact of high-oleic peanuts over the regular cultivar. However, in contradiction to this surmise, in the liver AMPK was upregulated merely by HFD and in adipose tissue by the combination of HFD and the traditional peanuts, while such induction was failed to be achieved by the addition of HO peanuts to HFD. Although the mechanism responsible for these AMPK activations is not comprehended it is tempting to suggest such activation, at least in the liver, is a consequence of energy shortage subsequent to mitochondrial damage due to fat accumulation. In such context, the absence of AMPK activation in the group supplemented with high-oleate peanuts represents a positive rather than a negative finding with the deduction of adequate energy metabolism in those groups. More studies are needed of course to corroborate this speculation.

Prolonged nuts consumption was also implicated in ameliorating oxidative stress (27). Minor liver inflammation was present in the HFD and HFDh groups, as evident by increased iNOS protein levels and SAA-1 expression in both groups as well as TNFα expression in the latter. A similar expression pattern of pro-inflammatory cytokines was found in adipose tissue. Essentially, in both tissues, HFD-induced inflammation was diminished by the incorporation of high-oleic peanuts into this diet. A recent work conducted on overweight men evaluated the ability of high-oleic peanuts daily consumption (56 gr/day for 4 weeks) combined with a hypocaloric diet to suppress inflammation and oxidative stress. Results fall short of expectation with no identification of such beneficial influences (28). Likewise, controversial results were also obtained from studies that investigated the mitigating effects of diverse nuts on oxidative stress and inflammation. These rebuttals appear to be derived from differences in fundamental parameters including, nut type and cultivar, nut dosage, and population feature of study design (e.g., study duration).

Intense, ongoing research effort is devoted to comprehensively defining the effects of diet on gut microbiota diversity and composition and the consequences of these interoceptive changes on human health. At the phylum level, the current study found a vigorous decrease and increase in Bacteroidetes and Firmicutes phyla, respectively, and consequently elevated Firmicutes/Bacteroidetes (F/B) ratio in all HFD-fed groups.

Alterations in those phyla in response to HFD feeding are well documented in the literature. A greater F/B ratio was measured in humans and rodents with obesity and related metabolic conditions, giving rise to the posit that this elevation represents a feature of obesity. Yet, this corollary is not compellingly supported by current data, and more research is required to elucidate the metabolic results driven by this increment or what accompanies it (29). Moreover, differences in this ratio between human and animal models of NAFLD were also elucidated (8), further questioning whether a general conclusion regarding its repercussion can be driven. Finally, whether and which of these modifications precede or proceed with metabolic and/or dysbiosis changes are yet to be utterly determined. Consist with this notion, the shift in F/B ratio appears to be, in the main part, attributed to the rise in the Clostridia class of the Firmicutes phyla. A recent study conducted by Petersen et al. (30) suggested Clostridia species diminishes lipid absorption and renders protection from obesity. Thus, the registered elevation in this class may represent a protecting or compensating mechanism aimed to tone down weight gain.

Concerning the observed increase in Proteobacteria in the groups that were fed with HFD with or without peanuts, although the augmented proliferation of this phyla is putative as pathogenic, its role here is hard to define. Several classes of Proteobacteria were found to be associated with intestinal and extra-intestinal diseases (31). Yet, except for Deltaproteobacteria, none of the other families were found to be significantly altered in the present study. Recent work by Apollo et al. suggested enriched levels of Deltaproteobacteria may constitute a part of the gut microbiota alignment to facilitate colonization resistance, a process that aims to impede infection, in previously infected mice. The corollary that arises from Apollo et al., work is that taurine utilization by Deltaproteobacteria alleviates their expansion and sulfide production by these bacteria to subsequently hamper pathogen respiration (32). Accordingly, the expansion of Deltaproteobacteria appears to confer beneficial outcomes under different conditions associated with “leaky-gut” like those occasionally documented in metabolic diseases (33). However, it should be stressed that colonic mucosal damage was also attributed to increased sulfide in individuals with ulcerative colitis (31).

At the family level, peanuts supplementation failed to restore or hinder the negative effect of HFD on the enrichment of the beneficial bacteria Bifidobacteriaceae, s24-7, and Verrucomicrobiaceae. Likewise, these additions were incompetent to inhibit the aberrant robust growth of metabolic diseases/alterations-associated bacteria of Lachnospiraceae and Desulfovibrionaceae families (34). Yet, interesting modifications were indeed registered in the gut microbiota of those groups that should be considered.

Both peanuts added groups demonstrated an adequate Deferribacteraceae and Clostridiaceae abundance despite the propensity increase and the profound decrease, respectively, detected in those families in the HFD-fed group. The enhanced presence of Deferribacteraceae (the only described family of the phylum Deferribacteres) was noted in animal models of obesity or diabetes (35). Data obtained from work conducted in rodents have implicated this phylum as being a part of the pathogenesis in inflammatory circumstances, conjecturing it encourages gut colonization during these conditions (35–37). Of great interest, a flourishment of the mucin-degrader Mucispirillum genus of this phylum was previously associated with gut pathology (36, 37). In the present study, Mucispirillum was the foremost genus of this phylum. Although the Mucispirillum genus did not portentously overgrow following the consumption of HFD, a tendency was disclosed. Essentially, the addition of peanuts to this diet efficaciously resulted in a marked reduction in the Mucispirillum genus.

The notion of imbuing gut barrier capacity following habitual peanuts addition to a HFD is further supported by differences in the levels of families with butyrate-producing/promoting bacteria (directly or indirectly),(38, 39) of Clostridiaceae, Prevotellaceae, Lachnospiraceae, and Ruminococcaceae (34). Whereas the levels of the last two families increased or tended to increase in all HFD treated groups, those of the first were downregulated in HFD-fed mice while remaining unaffected in those that were also supplemented with peanuts. Prevotellaceae presence was enriched merely in the HFDo group, which may imply an additional capacity for butyrogenesis in those mice. The role of butyrate in the gut is decidedly imperative and was shown to promote favorable metabolic and physiological effects, comprising enhanced insulin sensitivity and gut-wall integrity among others (7, 8, 40).

Although it is very tempting to propose these results are representative of a healthier gut in terms of mucus layer integrity in peanuts-fed mice, such an inference cannot yet to be made. Nonetheless, it appears that lower Mucispirillum genus (Schaedleri sp.) abundance along with enhanced butyrate-generation aptitude, observed in the groups that were fed with peanuts-added HFD, represents a consequential attempt to accommodate better barrier capacity under an obesogenic diet.

Two families, namely, Coriobacteriaceae and Erysipelotrichaceae of the Actinobacteria and Firmicutes phyla, respectively, were distinctively altered in the HFDo group where they displayed reduced relative amounts. Coriobacteriaceae can be considered as “pathobiont,” a term used to define symbiont who may potentially promote pathology, though only under specific circumstances (41–43). Previous evidence has demonstrated the existence of positive correlations between Coriobacteriaceae and hepatic TG concentrations in C3H/Orl female mice and plasma non-HDL levels in hamsters (43), converging with the notion that this family participates in the host bile acid and lipid metabolism (44). Notably, the role of diminished amounts of Coriobacteriaceae in the HFDo group is hard to determine. Of great interest, lower levels of Erysipelotrichaceaewere distinguished in the HFDo group. Accelerated levels of the Erysipelotrichaceae family were found in HFD-fed mice and humans as well as in mice models of liver injury and colitis-induced diabetes (44–46), thus arguing for the favorable effects of high-oleic peanuts addition to HFD.

## Conclusion

In summary, data presented here suggest the new high oleic acid cultivar is metabolically superior to the traditional peanut type and was associated with a better inflammatory state and microbial profile. Nevertheless, the glycemic response failed to be restored by peanuts addition and appeared to be negatively reinforced in those groups, predominantly in the traditional peanut type. Further research is essentially required to further define the impact of this new high-oleic cultivar and to gain a more in-depth insight into the precise underlying mechanisms.

## Data Availability Statement

The datasets presented in this study can be found in online repositories. The names of the repository/repositories and accession number(s) can be found below: https://www.ncbi.nlm.nih.gov/, All raw sequence data was uploaded to SRA under the BioProject accession number PRJNA784088.

## Ethics Statement

The animal study was reviewed and approved by the Institutional Animal Care Ethics Committee, The Hebrew University of Jerusalem.

## Author Contributions

SA-C: data interpretation and original draft preparation. NT-S: performing experiments and acquiring data. NT-S, GZ, RH, NS, and AN: data processing and analyzing. ZM, RH, and GZ: providing support in the experiments. ZM and NT-S: conceptualization and designing. All authors have read and agreed to the published version of the manuscript.

## Conflict of Interest

The authors declare that the research was conducted in the absence of any commercial or financial relationships that could be construed as a potential conflict of interest.

## Publisher’s Note

All claims expressed in this article are solely those of the authors and do not necessarily represent those of their affiliated organizations, or those of the publisher, the editors and the reviewers. Any product that may be evaluated in this article, or claim that may be made by its manufacturer, is not guaranteed or endorsed by the publisher.
