# Supplementary material for: Metabolic and Microbiome Alterations Following the Enrichment of a High-Fat Diet With High Oleic Acid Peanuts Versus the Traditional Peanuts Cultivar in Mice
Source: Front Nutr. 2022 Jun 15;9:823756. doi: 10.3389/fnut.2022.823756 (PMC9240694; doi:10.3389/fnut.2022.823756)
Supplement: Supplementary file 1 [file Image_1.pdf]

Supplementary materials:

<u>**Supplemental Fig. 1:**</u>

A

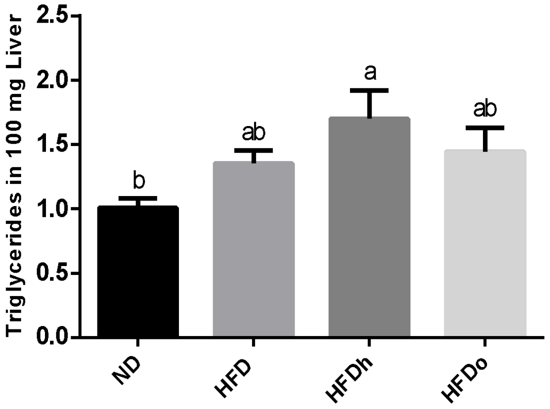

B

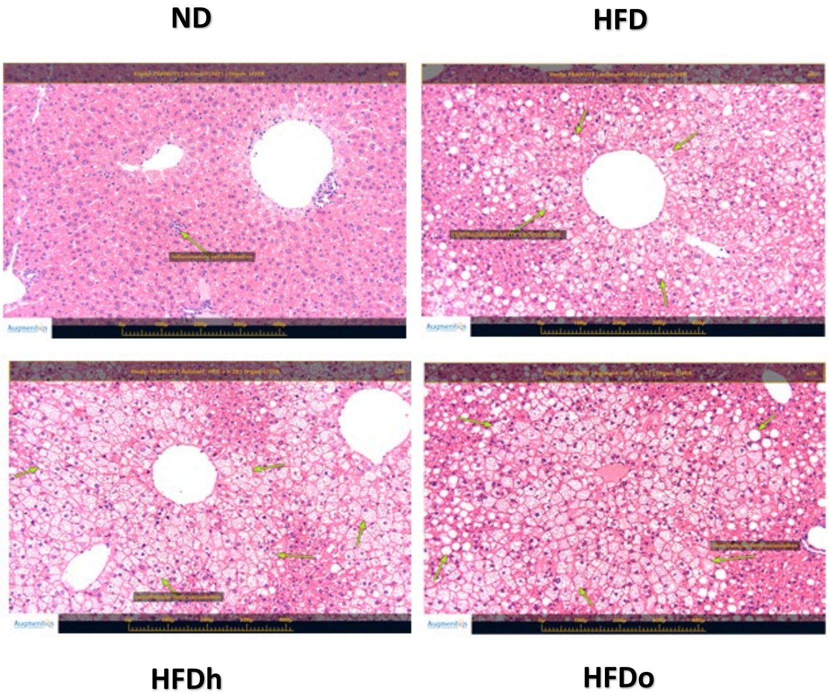

Figure S1. The effect of diets on triglycerides accumulation and histology in liver tissue. The mice consumed either a normal diet (ND), a high-fat diet (HFD), high-fat diet plus 4% (w/w) HN (HFDh) or HO (HFDo) peanuts for 18 weeks. (A)The amount of triglycerides was measured in 100mg of tissue; (B) Liver histology from the right lobe stained in H&E (magnification X 200), the green arrows indicate inflammatory cell infiltration.. A Tukey-Kramer HSD post hoc statistical test was performed. The value presented are mean ± SE (n=8). The value displayed are mean ± standard error. Columns marked with different letters indicate statistically significant variances at p<0.05.
